# Investigating the impact of structured reporting on the linguistic standardization of radiology reports through natural language processing over a 10-year period

**DOI:** 10.1007/s00330-023-10050-2

**Published:** 2023-08-05

**Authors:** Jan Vosshenrich, Ivan Nesic, Daniel T. Boll, Tobias Heye

**Affiliations:** Department of Radiology, University Hospital Basel, Petersgraben 4, 4031 Basel, Switzerland

**Keywords:** Radiology, Language, Standardization, Linguistics, Report

## Abstract

**Objectives:**

To investigate how a transition from free text to structured reporting affects reporting language with regard to standardization and distinguishability.

**Methods:**

A total of 747,393 radiology reports dictated between January 2011 and June 2020 were retrospectively analyzed. The body and cardiothoracic imaging divisions introduced a reporting concept using standardized language and structured reporting templates in January 2016.

Reports were segmented by a natural language processing algorithm and converted into a 20-dimension document vector. For analysis, dimensionality was reduced to a 2D visualization with t-distributed stochastic neighbor embedding and matched with metadata. Linguistic standardization was assessed by comparing distinct report types’ vector spreads (e.g., run-off MR angiography) between reporting standards. Changes in report type distinguishability (e.g., CT abdomen/pelvis vs. MR abdomen) were measured by comparing the distance between their centroids.

**Results:**

Structured reports showed lower document vector spread (thus higher linguistic similarity) compared with free-text reports overall (21.9 [free-text] vs. 15.9 [structured]; − 27.4%; *p* < 0.001) and for most report types, e.g., run-off MR angiography (15.2 vs. 1.8; − 88.2%; *p* < 0.001) or double-rule-out CT (26.8 vs. 10.0; − 62.7%; *p* < 0.001). No changes were observed for reports continued to be written in free text, e.g., CT head reports (33.2 vs. 33.1; − 0.3%; *p* = 1).

Distances between the report types’ centroids increased with structured reporting (thus better linguistic distinguishability) overall (27.3 vs. 54.4; + 99.3 ± 98.4%) and for specific report types, e.g., CT abdomen/pelvis vs. MR abdomen (13.7 vs. 37.2; + 171.5%).

**Conclusion:**

Structured reporting and the use of factual language yield more homogenous and standardized radiology reports on a linguistic level, tailored to specific reporting scenarios and imaging studies.

**Clinical relevance:**

Information transmission to referring physicians, as well as automated report assessment and content extraction in big data analyses, may benefit from standardized reporting, due to consistent report organization and terminology used for pathologies and normal findings.

**Key Points:**

• *Natural language processing and t-distributed stochastic neighbor embedding can transform radiology reports into numeric vectors, allowing the quantification of their linguistic standardization.*

• *Structured reporting substantially increases reports’ linguistic standardization (mean: − 27.4% in vector spread) and distinguishability (mean:* + *99.3* ± *98.4% increase in vector distance) compared with free-text reports.*

• *Higher standardization and homogeneity outline potential benefits of structured reporting for information transmission and big data analyses.*

**Supplementary Information:**

The online version contains supplementary material available at 10.1007/s00330-023-10050-2.

## Introduction

Many radiology departments have switched their reporting standard from free-text reporting to structured reporting templates in the last decades [1]. The use of such templates is increasingly advocated by many radiology societies [2, 3] and has been shown to be preferred by referring physicians given higher report completeness, clarity, and comprehensibility for the reader [4, 5]. This feedback from the radiologists’ target audience depicts one of the defined goals of structured reporting, which is to optimize information transmission with regard to the “Seven C’s of effective communication.” These are completeness, conciseness, consideration, concreteness, clarity, comparison, and correctness [6]. To reach this objective, reporting templates aim to convey information on pathology or absence thereof in a structured format to facilitate readability and information retrievability [7]. However, template organization, e.g., by means of organ-based subheadings, only represents the first step toward report standardization. To fully standardize radiology reports, the additional use of a specific lexicon or language, such as RadLex, is necessary [7, 8]. The implementation of reporting templates incorporating both factors may ultimately render radiology report content equally well standardized and machine readable like laboratory values or heart rate and blood pressure measurements are already today.

Given current trends toward big data analyses and the implementation of clinical data warehouses to facilitate data mining in clinical and in research settings, the aim to standardize radiology reports is of particular interest [7]. While there have been various approaches to assess and extract data from structured and non-structured radiology reports using natural language processing algorithms in specific scenarios, e.g., presence of pulmonary embolism or incidental pulmonary nodules [9, 10], only few investigations attempted to assess the effects of a transition from free-text reporting to structured reporting on a linguistic level [11].

The aim of our study was to investigate how the broad implementation of structured reporting templates and a reporting concept emphasizing factual standardized reporting language and discouraging ambiguous terminology and hedging in two radiology subspecialty sections in a university hospital setting affects radiology reporting language. We hypothesized that the combined use of structured templates and the advocated reporting concept would lead to higher linguistic standardization of distinct report types, better linguistic distinguishability between different types of reports, and higher reporting consistency compared to free-text reporting.

## Materials and methods

### Data sample

All radiology reports dictated between January 2011 and June 2020 in the body, cardiothoracic, musculoskeletal imaging, and neuroradiology divisions of our tertiary care radiology department were retrospectively included without preselection. Plain report text was used for analysis. Metadata of the reported imaging study, i.e., modality, examination type (e.g., CT abdomen/pelvis), imaging protocol, and date of the examination was available for each report.

### Reporting style

In January 2016, our department’s body and cardiothoracic imaging sections switched reporting routine from traditional free-text reports to structured reporting templates. Structured reporting in our case represents organized or itemized templates with headings and subheadings. Additionally, a reporting concept emphasizing factual standardized reporting language conveying certainty was implemented. For instance, the use of ambiguous language or expressions containing hedging statements, such as “prominent” or “accentuated,” was discouraged as it was shown to diminish certainty [12, 13]. Reporting templates were drafted by senior staff radiologists based on suggestions by the RSNA reporting initiative [3]. Drafts were jointly reviewed with referring physicians, and content was in some instances amended to account for clinicians’ preferences and needs specific to our institution. The templates’ findings section organization is either organ based with prepopulated normal findings (e.g., CT abdomen/pelvis), or feature based with a point-and-click approach when tailored to specific scenarios (e.g., rectal cancer staging MRI or run-off MR angiography). Examples are provided in the online supplement (figures S1 and S2).

In contrast to the body and cardiothoracic imaging sections, our musculoskeletal imaging division continued to report imaging studies in a non-structured free-text format. Finally, our neuroradiology section introduced structured reporting templates for some examinations (e.g., stroke CT) in 2019, but the majority of reports remains to be in a non-structured format. Data from these two subspecialty sections thus almost entirely consisted of free-text reports, while data from body and cardiothoracic imaging included both structured (2016–2020) and non-structured (2011–2015) radiology reports.

### Data processing

Plain report text of all available radiology reports was extracted from our institutional radiology information system (RIS).

In a first step, documents were divided into reports containing a separate “findings” and “impression” section (ultrasound, CT, and MRI studies) and reports consisting of a single combined “findings and impression” section (radiographs) using regular expressions. For reports containing distinct “findings” and “impression” sections, content was separated to allow for individual assessment of both report sections. This step was necessary, since the structured or itemized format of the newly introduced reporting templates is limited to the findings section. The impression section remains to be dictated in prose style, irrespective of the subspecialty section or template used. Without separation of report content into these two sections, assessment would have been biased, even though a distinct summary style and a format using bullet points to list primary and secondary/incidental findings prioritized by importance is advocated in our department. For reports with a combined “findings and impression” section, this step was not necessary, as the newly introduced structured reporting templates for these reports (e.g., chest radiographs) also consisted of a single section only. All report content outside these two sections, i.e., patient history and procedure information, was discarded.

In a second step, report content was converted into a 20-dimension number vector using the doc2vec approach [14]. This unsupervised neural network-based technique learns continuous distributed representations for documents, taking into account the text’s semantics and word order to obtain a numeric representation of a document, or, in our case, the distinct sections of a radiology report. Its concept is based on the word2vec model, which was introduced in 2013 and is used to vectorize words within a document [15]. There, the vector distance of two distinct words in vector space represents their similarity in meaning based on context. For example, the words “strong” and “powerful” would be close together in vector space, while the vector distance between the words “strong” and “weak” would be relatively far. Instead of averaging vectors for each word in a document, the doc2vec approach incorporates an additional document vector, intending to represent the document’s concept. This is known to outperform simple-averaging of word vectors [14]. Text data was tokenized using the Natural Language Toolkit library and each document was tagged with a unique identifier. Models were initialized with a vector size of 20, an initial learning rate of 0.025, and a minimum word frequency of 1. Training was performed over 50 epochs, with the learning rate linearly decreasing at each epoch by 0.0002. Model vocabulary was constructed using the tagged data. Document and word vectors were iteratively updated through training. Upon completion, the trained models were used to infer vector representations for each document in the dataset.

In a third step, the 20-dimension document vector had to be reduced to two dimensions to allow for data plotting, visualization, and statistical analysis. We used t-distributed stochastic neighbor embedding (t-SNE) to aggregate all dimensions into two [16]. In contrast to principal component analysis, t-SNE is a non-linear dimensionality reduction technique which preserves the local structure (neighborhood) of data and is substantially less affected by outliers.

### Visualization and statistical analysis

Two-dimensional data was visualized using commercially available software (Tableau 2022.1, Tableau Inc.). To facilitate visualization, data was prepared in 2-year sets (e.g., 2014 vs. 2019) with projections of 10,000 documents per year and subspecialty section. These were joined with RIS metadata (i.e., year, imaging modality and imaging protocol). Document vectors were visualized as scatter plots with RIS attributes used for color coding.

**Fig. 1:**
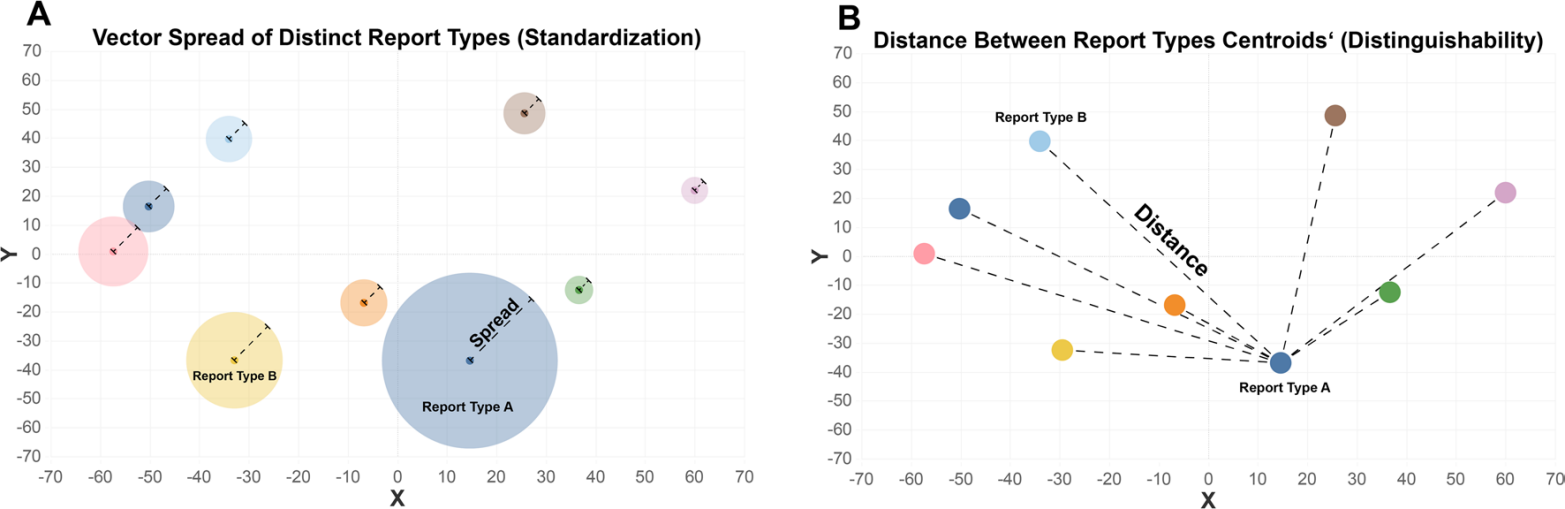
Schematic drawings how report standardization (**A**) and distinguishability (**B**) were assessed. Linguistic standardization (**A**) is represented by the spread (= standard deviation) of distinct radiology report types (expressed by color coding) around their centroid (= mean) in vector space. Less spread equals higher document similarity (thus higher standardization of a distinct report type). Distinguishability (**B**) between distinct types of radiology reports (expressed by color coding) is represented by the distance between their centroids in vector space. A higher distance between two centroids equals lower document type similarity (thus better distinguishability of the two report types)

To assess the effects of structured reporting on reporting language standardization for distinct types of imaging studies, the document vector spread around their centroid was calculated separately for free-text reports and structured reports (Fig. 1a). Standard deviations were then compared between the two reporting standards using an *F*-test. Distinguishability of radiology reports between distinct imaging protocols was assessed by comparing the distance between the document vectors’ centroids before and after the introduction of structured reporting templates (Fig. 1b). Reporting consistency following the introduction of structured reporting templates was assessed through comparison of centroid location and vector spread between distinct years following the change in reporting standard (e.g., 2018 vs. 2019). *p* < 0.05 was considered to represent a statistically significant difference.

## Results

### Data sample

**Fig. 2:**
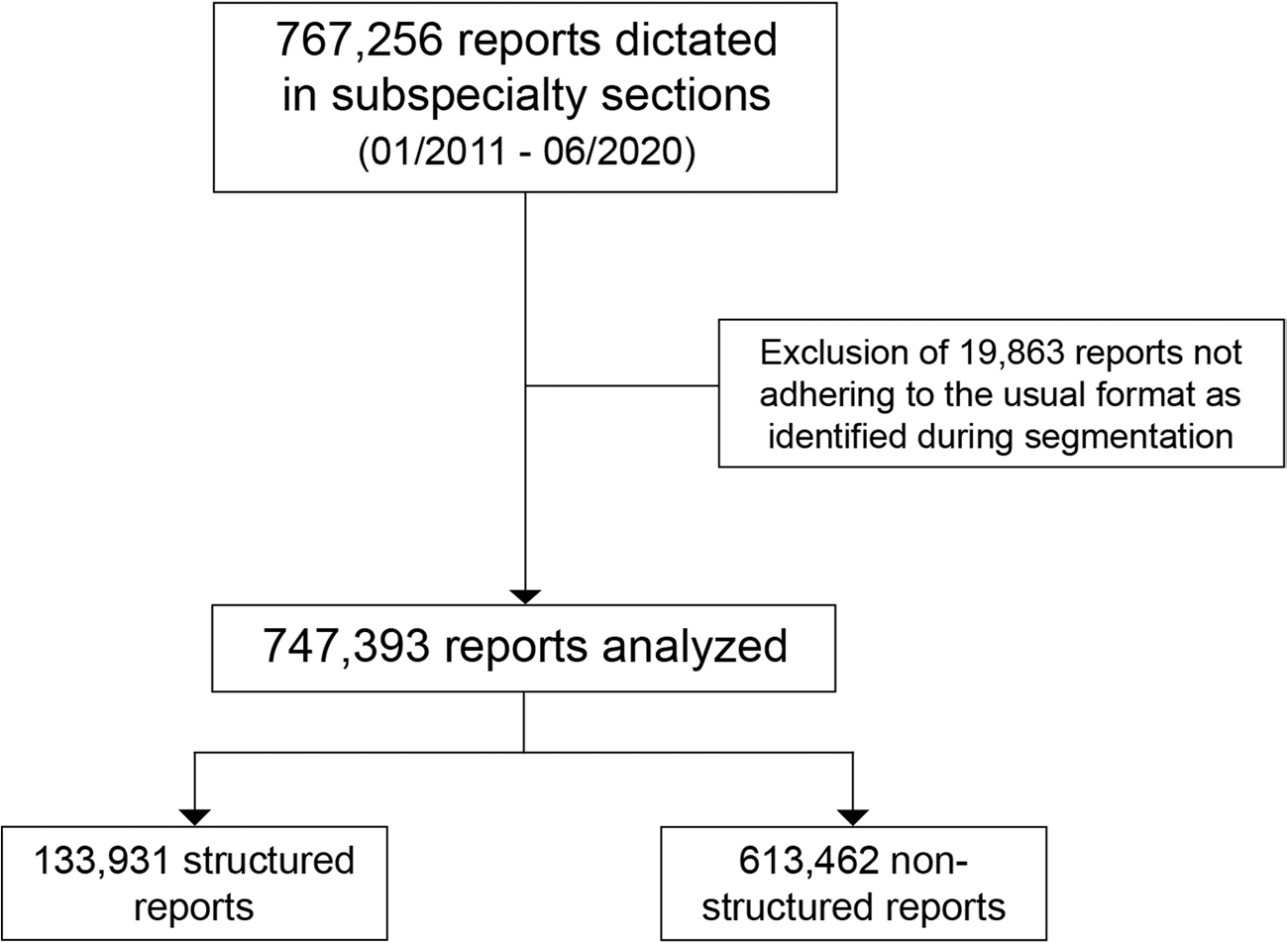
Flowchart of the study sample

A total of 767,256 radiology reports dictated between January 2011 and June 2020 were retrieved from the RIS. In 19,863 instances, report segmentation through regular expressions failed because report content did not adhere to the expected report sections (“findings,” “impression,” or “findings and impression”). These reports were excluded from further analysis, resulting in a final study sample of 747,393 reports. Data comprised of 133,931 (17.9%) structured reports and 613,462 (82.1%) non-structured reports. A flowchart of the study sample is visualized in Fig. 2; data distribution is provided in Table 1.

**Table 1:** Distribution of radiology reports among subspecialty sections and reporting standard

Subspecialty section	Non-structured reports	Structured reports	Total
Body	77,129 (10.3)	43,535 (5.8)	120,664 (16.1)
Cardiothoracic	102,266 (13.7)	85,596 (11.5)	187,862 (25.1)
Musculoskeletal	287,055 (38.4)	n/a	287,055 (38.4)
Neuroradiology	147,012 (19.7)	4800 (0.6)	151,812 (20.3)
Total	613,462 (82.1)	133,931 (17.9)	747,393 (100)

Data are numbers of radiology reports, with percentages in parentheses. Percentages are based on the 747,393 reports and are rounded to the first decimal place

n/a = not available

### Linguistic similarity of distinct radiology report types

**Fig. 3:**
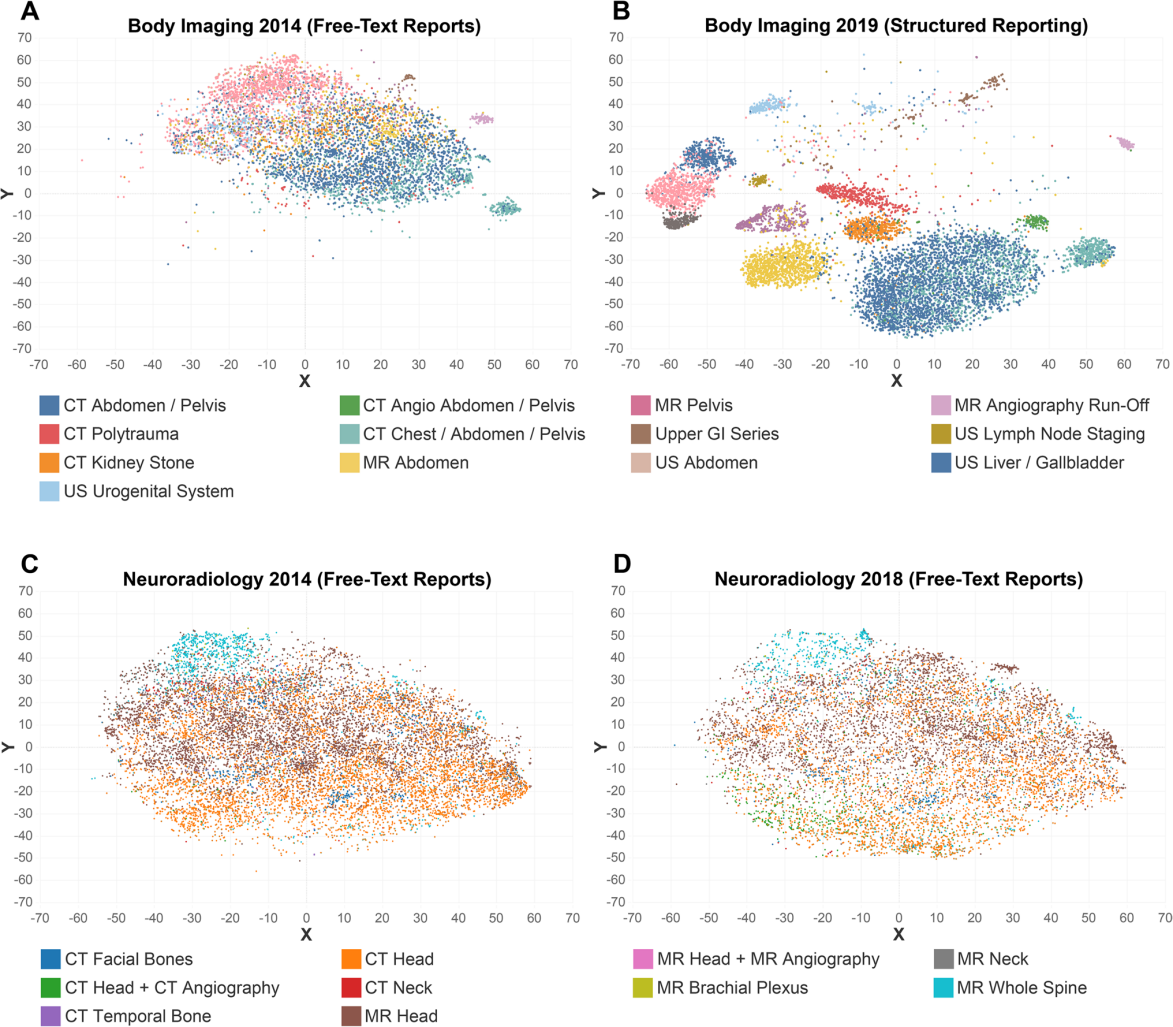
Distribution of radiology reports in vector space before (**A**) and after (**B**) the introduction of structured reporting in body imaging. Distinguishability and clustering of distinct radiology report increased with structured templates compared with overlapping data points for free-text reporting. Neuroradiology report distribution remained unchanged between 2014 (**C**) and 2018 (**D**), given continued free-text format reporting

With structured reporting, document spread around the centroids decreased overall and for most distinct radiology report types in the body and cardiothoracic imaging divisions. This means that document vectors of a specific report type (e.g., all kidney stone CT reports) were closer together in vector space and document variation among this specific report type decreased (Figs. 1a and 3).

**Fig. 4:**
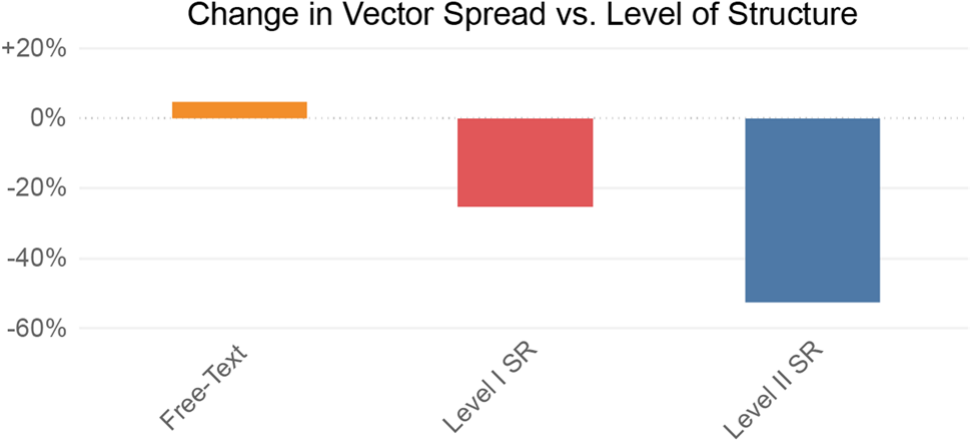
Comparison of changes in vector spread between 2014 and 2019 for different levels of reporting. Level I structured reports represent templates with a structured layout; level II represents templates with a structured content [7]. SR = structured report

When comparing the newly structured reports from 2019 to free-text reports from 2014 in the body and cardiothoracic imaging sections, an overall decrease in vector spread of − 27.4% (21.9 [2014] vs. 15.9 [2019]; *p* < 0.001) was observed. This was true for most distinct report types from body imaging, including both highly structured templates using a point-and-click approach, e.g., run-off MR angiography (15.2 vs. 1.8; − 88.2%; *p* < 0.001), as well as reports with organ-based subheadings and prepopulated normal findings, e.g., polytrauma CT (23.2 vs. 10.5; − 54.7%; *p* < 0.001) or kidney stone CT reports (18.1 vs. 11.3; − 37.6%; *p* < 0.001). Body imaging reports are visualized in Fig. 3a, b. Similar decreases were observed for structured reports in the cardiothoracic imaging division, e.g., for double-rule-out CT (26.8 vs. 10.0; − 62.7%; *p* < 0.001), cardiac MRI (17.8 vs. 13.4; − 24.7%; *p* < 0.001), and supine chest radiographs (28.7 vs. 21.1; − 26.5%; *p* < 0.001). In both imaging divisions, decreases in vector spread were higher for level II structured reports compared with level I structured reports (− 53.4% vs. 25.4%; *p* < 0.001; Fig. 4).

For reports from the musculoskeletal and neuroradiology sections, who continued to report imaging studies in a non-structured free-text format, no decreases in document spread were observed between 2014 and 2019 (musculoskeletal imaging) or 2014 and 2018 (neuroradiology; the year was amended to 2018 to avoid bias since some structured reporting templates were introduced in July 2019 as noted in “Materials and methods”), e.g., CT head (33.2 vs. 33.1; − 0.3%; *p* = 1) or CT facial bones (30.6 vs. 30.5; − 0.3%; *p* = 0.96). In some instances, document spread around the centroid even increased (thus, similarity further decreased), e.g., for whole spine MRI reports (37.8 vs. 43.4; + 14.8% *p* < 0.001). Neuroradiology reports are visualized in Fig. 3c, d. Detailed data for all report types including *p* values is provided in Tables 2 and 3.

**Table 2:** Comparison of document spread around their centroids (= standard deviation) and distance of report types’ centroids to a reference point in vector space following t-distributed stochastic neighbor embedding (t-SNE) before (2014) and after (2019) the introduction of structured reporting

Report type	Spread 2014 (FTR)	Spread 2019 (SR)	Difference	*p* value	Distance 2014 (FTR)	Distance 2019 (SR)	Difference
CT chest	24.5	23.9	− 2.5%	0.28	–^c^	–^c^	–^c^
CT coronary angiography	29.1	21.7	− 25.4%	0.01	48.1	81.5	69.4%
CT pulmonary angiography	27.5	25.0	− 9.1%	0.001	35.9	47.2	+ 31.5%
CT double-rule-out	26.8	10.0	− 62.7%	< 0.001	46.6	48.5	+ 4.1%
CT chest-abdomen-pelvis^a^	21.5	20.4	− 5.1%	0.02	14.4	8.8	− 38.9%
CT chest-abdomen-pelvis^b^	32.5	13.1	− 59.7%	< 0.001	38.9	54.7	+ 40.6%
CT abdomen-pelvis	18.0	17.9	− 0.6%	1	–^c^	–^c^	–^c^
CT kidney stone	18.1	11.3	− 37.6%	< 0.001	11.0	14.3	+ 30.0%
CT polytrauma	23.2	10.5	− 54.7%	< 0.001	8.8	42.3	+ 380.7%
Cardiac MRI	17.8	13.4	− 24.7%	< 0.001	28.8	63.9	+ 121.9%
MRI chest	21.3	18.4	− 13.6%	0.36	10.1	29.4	+ 191.1%
MRI abdomen	19.5	15.9	− 18.5%	< 0.001	13.7	37.2	+ 171.5%
MRI pelvis	19.0	13.4	− 29.5%	< 0.001	20.4	49.5	+ 142.7%
MRI angiography run-off	15.2	1.8	− 88.2%	< 0.001	30.5	80.7	+ 164.6%
Upper GI series	22.8	14.7	− 35.5%	< 0.001	23.1	82.5	+ 257.1%
Abdominal ultrasound	17.2	11.0	− 36.0%	< 0.001	37.7	77.7	+ 106.1%
Lymph node staging ultrasound	17.1	10.6	− 38.0%	< 0.001	40.0	64.7	+ 61.8%
Liver ultrasound	17.6	10.1	− 42.6%	< 0.001	35.8	80.9	+ 126.0%
Urogenital ultrasound	15.0	14.7	− 2.0%	0.64	34.1	86.7	+ 154.3%
Chest radiographs (upright)	28.1	28.1	− 0%	1	–^c^	–^c^	–^c^
Chest radiographs (supine)	28.7	21.1	− 26.5%	< 0.001	13.3	29.3	+ 120.3%
Overall structured reporting	21.9	15.9	− 27.4%	< 0.001	27.3	54.4	+ 99.3%

*FTR* free-text reports, *SR* structured reports

^a^Data from body imaging

^b^Data from cardiothoracic imaging

^c^Report types marked with an asterisk served as reference points for distance measurements

**Table 3:** Comparison of document spread around their centroids (= standard deviation) and distance of report types’ centroids to a reference point in vector space following t-distributed stochastic neighbor embedding (t-SNE) for report types continued to be written in free-text style

Free-text report type	Spread 2014	Spread 2019^c^	Difference	*p* value	Distance 2014	Distance 2019^c^	Difference
CT facial bones	30.6	30.5	− 0.3%	0.96	9.8	9.8	+ 0%
CT head	33.2	33.1	− 0.3%	1	–^c^	–^c^	–^c^
CT neck	28.6	36.4	+ 27.3%	0.01	35.7	28.7	− 19.6%
CT temporal bone	28.7	27.4	− 3.5%	0.88	26.0	34.7	+ 33.5%
MRI head	31.1	32.6	+ 4.8%	1	18.2	21.1	+ 15.9%
MRI head + MRA	30.3	35.0	+ 15.5%	0.94	28.3	11.1	− 60.8%
MRI neck	23.7	26.6	+ 12.1%	0.09	41.7	38.8	− 7.0%
MRI brachial plexus	25.5	24.4	− 4.3%	0.83	46.2	51.4	+ 11.3%
MRI whole spine	37.8	43.4	+ 14.8%	< 0.001	36.9	19.0	− 48.5%
MRI shoulder	32.5	37.1	+ 14.2%	0.16	–^c^	–^c^	–^c^
MRI lumbar spine	33.3	30.4	− 8.7%	0.03	58.5	69.6	+ 19.0%
MRI cervical spine	34.5	30.2	− 12.5%	< 0.001	56.6	66.9	+ 18.2%
MRI hip	33.1	40.6	+ 22.7%	0.06	12.6	15.2	+ 20.6%
MRI wrist	35.6	34.0	− 4.5%	0.74	12.4	14.5	+ 16.9%
MRI knee	32.5	33.2	+ 2.2%	0.58	12.8	8.8	− 31.3%
MRI ankle	34.7	33.7	− 2.9%	0.65	9.2	10.5	+ 14.1%
CT pelvis	31.6	33.1	+ 4.7%	0.62	25.6	31.4	+ 22.7%
Overall free-text reporting	31.6	33.0	+ 4.4%	0.85	28.7	28.8	+ 0.3%

^c^Report types marked with an asterisk served as reference points for distance measurements

### Distinguishability between different radiology report types

With structured reporting templates, vector distances between the report types’ centroids increased in the body and the cardiothoracic imaging divisions (mean: 27.3 [2014] vs. 54.4 [2019]; + 99.3 ± 98.4%). This means that different report types (e.g., CT abdomen/pelvis report vs. liver ultrasound report) can be distinguished visually and statistically, as they are distributed among the coordinate system and document vectors of different report types do not overlap (Figs. 1b and 3a, b). When using the centroid of CT abdomen/pelvis reports as a reference point for body imaging reports, mean vector distance to the other report types’ centroids increased by 141.2 ± 111.5% with structured reporting, e.g., CT abdomen/pelvis vs. CT polytrauma (8.8 [2014] vs. 42.3 [2019]; + 380.7%), CT abdomen/pelvis vs. MR abdomen (13.7 vs. 37.2; + 171.5%), and CT abdomen/pelvis vs. abdominal ultrasound (37.7 vs. 77.7; + 106.1%).

**Fig. 5:**
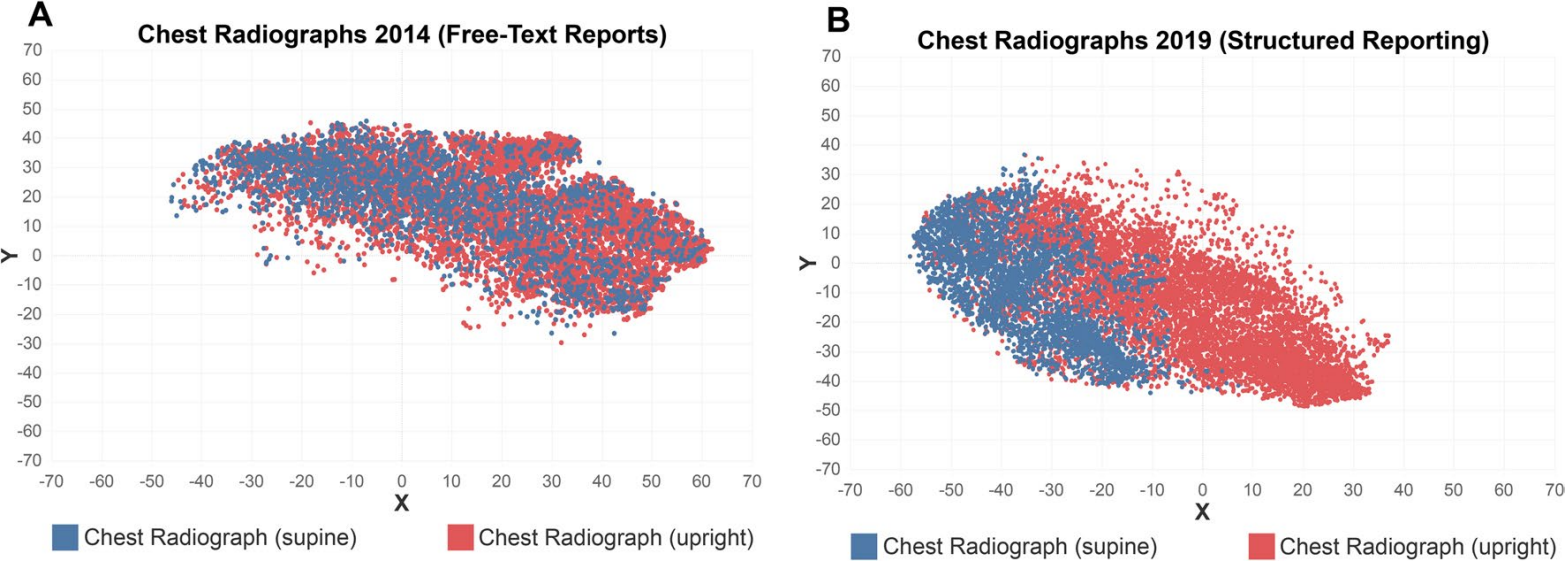
Distribution of chest radiograph reports in vector space before (**A**) and after (**B**) the introduction of structured reporting templates in cardiothoracic imaging, demonstrating higher distinguishability and clustering when reported with dedicated structured reporting templates

Similar results were observed for reports from cardiothoracic imaging. With chest CT reports serving as reference point, mean vector distance to other report types increased by 67.6 ± 74.5%, e.g., CT chest vs. cardiac MRI (28.8 vs. 63.9; + 121.9%) and CT chest vs. MRI chest (10.1 vs. 29.4; + 191.1%). This was also true when comparing upright and supine chest radiograph reports (13.3 vs. 29.3; + 120.3%). Increases in vector distance between distinct report types are visualized in Fig. 3a, b for body imaging and in Fig. 5 for chest radiographs.

For free-text reports from the musculoskeletal and neuroradiology sections, overall vector distance remained unchanged (28.7 vs. 28.8; + 0.3 ± 27.2%). For distinct report types, the changes were variable without a clear trend. Vector distances remained unchanged in some instances, e.g., CT head vs. CT facial bones (9.8 vs. 9.8; + 0%); decreased in some instances, e.g., CT head vs. CT neck (35.7 vs. 28.7; − 19.6%); or mildly increased in some instances, e.g., CT head vs. MRI head (18.2 vs. 21.1; + 15.9%). Detailed data for all report types is provided in Tables 2 and 3.

### Reporting consistency with structured reporting templates

Comparison of distinct years with sole use of structured reporting templates (e.g., 2018 vs. 2019) showed that document vectors spread remained lower than with free-text reporting or decreased even further (16.7 [2018] vs. 15.5 [2019]; *p* = 0.06). In body imaging, this was true, e.g., for CT chest-abdomen-pelvis (27.0 [2018] vs. 27.0 [2019]; − 0.1%; *p* = 0.95) and abdominal ultrasound reports (15.3 vs. 14.8; − 3.1%; *p* = 0.38). The continuously lower vector spread over several years thus represents a consistently higher similarity between individual documents of the same report type. With the continuous use of structured reporting templates, vector spread decreased even further over the years in some instances, e.g., CT polytrauma (16.2 vs. 13.2; − 18.6%; *p* < 0.001), run-off MR angiography (11.5 vs. 2.5; − 78.2%; *p* < 0.001), and lymph node staging ultrasound (27.1 vs. 23.8; − 52.8%; *p* < 0.001). Data for report types including *p* values is summarized in Table 4.

**Table 4:** Comparison of document spread around their centroids (= standard deviation) in vector space following t-distributed stochastic neighbor embedding between 2018 and 2019 following the introduction of structured reporting templates in the body imaging division

Structured report type	Spread 2018	Spread 2019	Difference	*p* value
CT abdomen-pelvis	23.3	24.2	+ 4.1%	0.06
CT chest-abdomen-pelvis	27.0	27.0	− 0.1%	0.95
CT kidney stone	16.0	15.1	− 5.6%	0.21
CT polytrauma	16.2	13.2	− 18.6%	< 0.001
CT angiography abdomen-pelvis	23.7	23.6	− 0.2%	0.98
MRI abdomen	14.9	16.4	+ 9.8%	0.004
MRI pelvis	14.9	12.9	− 13.8%	0.002
MRI angiography run-off	11.5	2.5	− 78.2%	< 0.001
Upper GI series	7.1	8.1	+ 13.8%	0.13
Abdominal ultrasound	15.3	14.8	− 3.1%	0.38
Lymph node staging ultrasound	27.1	23.8	− 52.8%	< 0.001
Liver ultrasound	10.8	11.1	+ 3.1%	0.52
Urogenital ultrasound	9.1	9.0	− 0.3%	0.96
Overall	16.7	15.5	− 7.3%	0.06

**Fig. 6:**
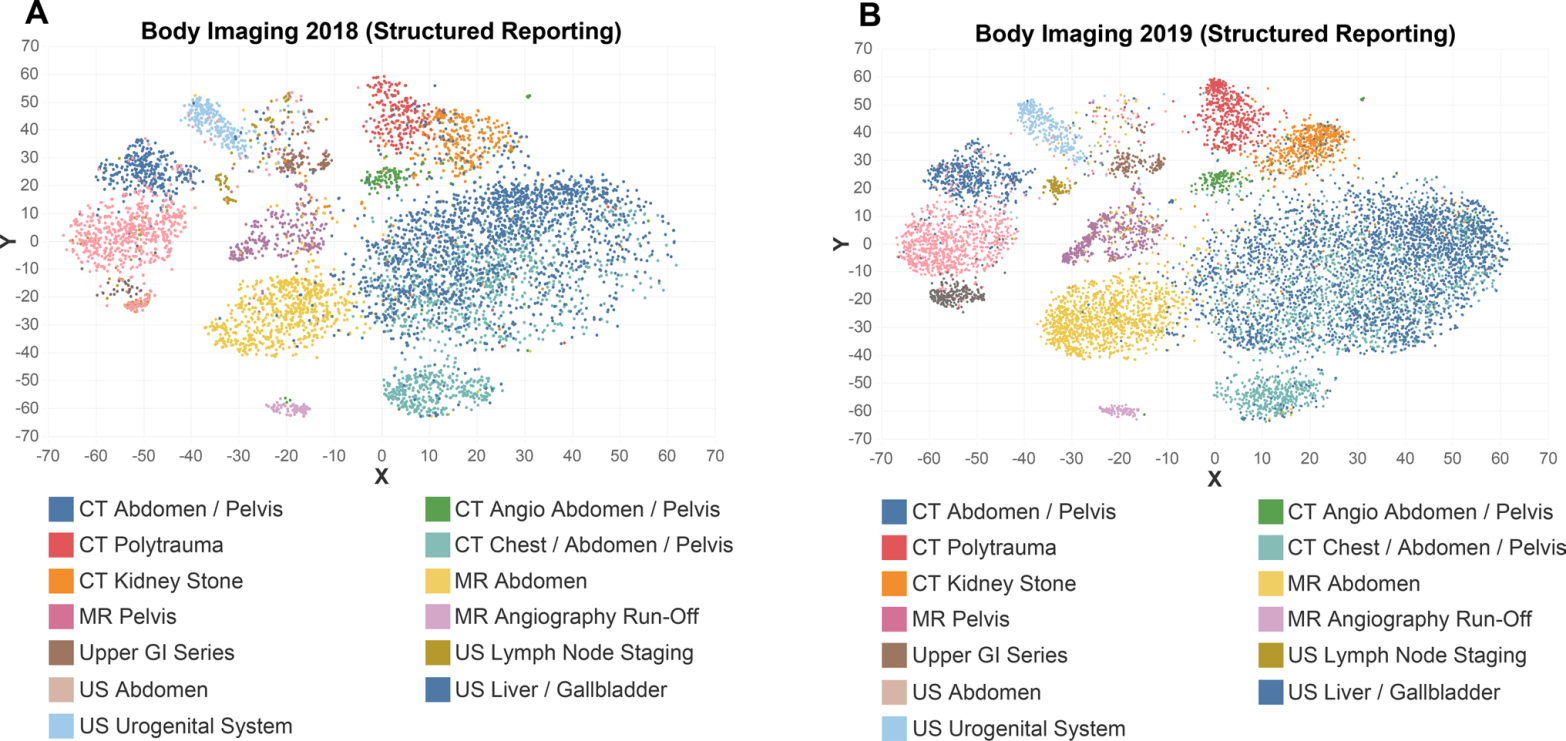
Distribution of radiology reports in vector space in 2018 (**A**) and 2019 (**B**) following the introduction of structured reporting templates. Centroid location in vector space and spread around the distinct report types’ centroids remain similar with structured reporting, indicating high reporting consistency

The location of report types’ centroids within the coordinate system remained almost similar between distinct years of structured reporting. When comparing 2018 and 2019, the mean overall difference in the centroids’ vector locations was 4.5 ± 4.4. Lowest changes in centroid location were observed for pelvic MRI (0.9), upper GI series (0.7), and urogenital ultrasound reports (0.3). The distinguishability between distinct report types thus remained high over several years of structured reporting, as the report type locations remained almost unchanged. Comparison of report distribution in vector space between 2018 and 2019 is visualized in Fig. 6. Evolution of report distribution in vector space over the course of 6 years is depicted in the online supplement (figure S3).

## Discussion

The aim of our study was to investigate whether the transition from traditional free-text reporting to structured reporting templates and the implementation of a reporting concept emphasizing factual standardized terminology in our tertiary care radiology department have affected reporting language. We observed significantly higher linguistic similarity, thus standardization, for most radiology report types following the introduction of structured templates, expressed by a mean decrease in document spread of − 27.4% (21.9 [2014] vs. 15.9 [2019]; *p* < 0.001). Similarly, the linguistic distinguishability of different report types, expressed by the distance between the distinct report types’ centroids in vector space, increased with structured reporting by 99.3 ± 98.4% on average (body imaging: + 141.2 ± 111.5% and cardiothoracic imaging: + 67.6 ± 74.5%). Finally, reporting consistency between distinct years of structured reporting was high, as document spread remained lower or decreased even further compared to free-text reporting (16.7 [2018] vs. 15.5 [2019]; *p* = 0.06), and centroid locations of the distinct report types within the coordinate system remained almost unchanged.

Several studies demonstrated higher report completeness and consistency when structured reporting templates are used [17–19]. The existing literature on this topic, however, mostly comprises of qualitative assessments of single reporting templates, e.g., comparing the number of features relevant for surgical planning mentioned in structured and non-structured reports [19–21], and not providing a macroscopic view on how a change in reporting standard affects radiology reporting language in general. Our results from a natural language processing analysis of 747,393 radiology reports show an increase in linguistic standardization and distinguishability of distinct types of radiology reports when dedicated reporting templates and factual language are used consistently in clinical routine. These increases translate into both a more homogenous and distinct language, e.g., to describe normal findings [22], as well as a consistent format used for specific imaging examinations when reported in a predefined manner using structured templates.

Our data included structured reports of different levels, i.e., reports with standardized layouts and prepopulated normal findings, where content editing by the reporting radiologist however remains to be possible, as well as reports with standardized content, where reporting can only be performed by choosing predefined items from drop-down menus or a point-and-click approach. The latter of these two levels is considered to be the most advantageous in terms of report standardization, given the predefined way of interaction with each item within the reporting template [7, 23]. Voices in the radiology community, however, repeatedly expressed concerns with regard to highly structured reporting templates in recent years, as they might negatively impact productivity, disrupt radiologists’ search patterns, or lead to constrained thinking during the reporting process when not being able to phrase how findings are related to each other [8, 24, 25]. While we did not assess report content accuracy in this study, our results are consistent with the assumption, that higher levels of structure within the reporting templates also lead to higher report standardization. This is well depicted by the observation, that reporting templates using an itemized point-and-click approach had the highest decrease in vector spread of all report types in our study, e.g., an 88.2% decrease for run-off MR angiography reports (15.2 [free-text reporting] vs. 1.8 [structured reporting]; *p* < 0.001).

Higher report standardization, distinguishability, and consistency may translate into lower variance between reports of different reporting radiologists or even different institutions. This is of importance, both in clinical and research settings. With regard to clinical routine, lower report variance can improve readability and comprehensibility for the recipients of radiology reports [4, 5, 26]. Furthermore, effective communication is needed to meaningfully influence behavior of radiology report recipients [27]. Several studies already investigated referring physicians’ preferences, and found a majority of them to favor structured reports for clinical decision-making due to higher levels of completeness and comprehensibility [28–30]. Also, the use of heterogenous terminology may lead to miscommunication, e.g., with regard to reporting doubt or certainty, as some expressions are perceived differently by radiologists and clinicians [30–32]. The use of standardized language therefore may help to avoid misunderstandings, reduce the necessity of addition clarification, and improve patient care.

In addition to potential benefits in clinical routine, standardized reports may also ease workflows in research scenarios. With current trends toward the establishment of data warehouses for single- or multicentric big data analyses, it is crucial to structure or standardize the content of radiology reports. Even though several studies provided evidence that natural language processing algorithms are also able to identify relevant information on the presence of specific pathologies in free-text reports [9, 10, 33], the consistent use of structured reporting templates and a standardized terminology may render this, often time-consuming, step in data preparation redundant.

Our study has limitations. The doc2vec model is only one of several existing methods used for natural language processing and incorporates vectors for both words and document structure. Therefore, reporting templates with prepopulated normal findings may be intrinsically closer together in vector space, given that a portion of this prepopulated terminology remain constant among most reports. This, however, could also be considered an advantage of structured reporting templates, since with new therapeutic approaches, especially in complex pathologic conditions involving multiple organ systems, radiologists are demanded to specifically mention pertinent negative findings in their reports by referring physicians. While a higher level of report standardization is thought to improve readability, comprehensibility, and accuracy, we did not assess radiology reports by qualitative means. We can therefore only assume that presenting the information gathered by a specific imaging study by means of a structured report using factual language improves information transmission to the referring physician.

In conclusion, we demonstrated that the combined use of structured reporting templates and factual language decreases report variance and increases report homogeneity on a linguistic level, likely tailored to specific reporting scenarios. Information transmission to referring physicians in clinical routine, as well as automated report assessment and content extraction in big data analyses, may benefit from the implementation of these concepts, due to consistent report organization and terminology used for both pathologies and normal findings.

### Supplementary Information

Below is the link to the electronic supplementary material.

Supplementary file1 (PDF 1750 KB)

## Abbreviations

CT: Computed tomography
ESR: European Society of Radiology
MRI: Magnetic resonance imaging
RIS: Radiology information system
RSNA: Radiological Society of North America
t-SNE: T-distributed stochastic neighbor embedding
US: Ultrasound

## Declarations

### Guarantor

The scientific guarantor of this publication is Jan Vosshenrich.

### Statistics and biometry

No complex statistical methods were necessary for this paper.

### Study subjects or cohorts overlap

Study subjects or cohorts have not been previously reported.

### Methodology

• retrospective

• experimental

• performed at one institution
