# Supplementary material for: Investigating the impact of structured reporting on the linguistic standardization of radiology reports through natural language processing over a 10-year period
Source: Eur Radiol. 2023 Aug 5;33(11):7496–506. doi: 10.1007/s00330-023-10050-2 (PMC10598161; doi:10.1007/s00330-023-10050-2)
Supplement: Supplementary file 1 — Supplementary file1 (PDF 1750 KB) [file 330_2023_10050_MOESM1_ESM.pdf]

Supplementary Material

Technique
Siemens 3T SkyraFIT MRI. 11 ml Primovist.

Findings
Compared to previous exam from 06/01/2014.

The visualized lower lungs and mediastinal structures don't show any abnormalities, as far as assessable by this modality. Nodular contour, macronodular appearance and hypertrophied caudate lobe as signs of liver cirrhosis. No signal drop or elevation of the liver parenchyma between in- and opposed phase, thus no evidence of steatosis or iron accumulation within the liver.
Inhomogeneous enhancement of the liver parenchyma, however no suspicious or hyperenhancing lesions in the arterial phase. No washout in the delayed phase. Unchanged non-enhancing defect in segment IVa following TACE procedure due to hepatocellular carcinoma. Mild reactive surrounding hyperemia of the parenchyma, similar to previous examinations. Scattered small T2 hyperintense, T1 hypointense, non-enhancing and benign appearing lesions throughout the liver, probably simple cysts. No diffusion restrictions. Mildly enlarged portal vein without evidence of thrombus. Recanalized umbilical vein as well as perigastric and esophageal varices as signs of portal hypertension.
Gallbladder and bile ducts unremarkable. No evidence of stones or an obstructing lesion.
Borderline size of the spleen with a maximum diameter of 13 cm. Known small T2 hyperintense, enhancing hemangioma of 1.5 cm near the upper pole (Se 4, Im 8).
Mild fatty atrophy of the pancreas. No signs of pancreatic duct obstruction. Mildly enlarged left adrenal gland without focal nodularity, normal appearing right adrenal gland. The visualized portions of both kidneys are unremarkable. No hydronephrosis. No focal or diffuse thickening of the gastrointestinal tract. No bowel obstruction. Mild perihepatic and perisplenic ascites.
Moderate arteriosclerosis. Normal diameter of the abdominal aorta. No high-grade stenosis of the abdominal vessels. No enlarged lymph nodes in the abdomen. The largest lymph node has a short axis diameter of 6 mm and is located in the hepatoduodenal ligament.
Mild degenerative changes of the thoracic and lumbar spine. Old, unchanged Th10 compression fracture. Small periumbilical hernia with protrusion of omental fat. Otherwise unremarkable soft tissues.

Impression
- No new HCC-suspicious liver lesions.
- Unchanged defect in liver segment IVa following TACE-treatment of an HCC.
- Signs of portal hypertension and mild ascites in the upper abdomen.
- Known old Th10 compression fracture.

____
LI-RADS NC = not categorizable due to image degradation or omission
LI-RADS M = probably / definite malignancy not HCC specific
LI-RADS TIV = definite tumor in vein
LI-RADS 5 = definitely HCC
LI-RADS 4 = probably HCC
LI-RADS 3 = intermediate probability of malignancy
LI-RADS 2 = probably benign
LI-RADS 1 = definitely benign observation

Reference
http://www.acr.org/quality-safety/resources/LIRADS

Technique
Siemens 3T Skyra MRI. 12 ml Primovist.

Findings
No comparison exams.

Liver:	Liver volume 1800 ml. Features of liver cirrhosis. Fat fraction 9.4% (normal: < 5%), no increased iron deposition: R2* 56/sec (normal: < 69/sec ± 22 at 3T).
Focal liver lesions:	1. 3.8 x 2.5 cm (Se 12, Im 8), segment VIII, arterial hyperenhancement, washout, enhancing capsule, diffusion restriction (LI-RADS 5)<br>2. 2.1 x 1.1 cm (Se 12, Im 17) segment VI, arterial hyperenhancement, washout, no enhancing capsule, diffusion restriction (LI-RADS 5)
Portal vein:	No portal vein thrombosis.
Portosystemic collaterals:	Extensive portosystemic collaterals with lienorenal shunt.
Arterial vessels:	Variant anatomy with left hepatic artery originating from the SMA.
Bile system:	Cholecystectomy. No bile duct dilatation. Normal biliary contrast excretion.
Spleen:	Splenomegaly (15 cm). No focal lesions.
Peritoneum:	Ascites in all four quadrants. No peritoneal lesions.
Pancreas:	Normal parenchyma. Normal dimensions of the pancreatic duct.
Adrenal glands:	Normal in size. No focal lesions.
Lymph nodes:	No lymphadenopathy in the upper abdomen.
Blood vessels:	Arterial and venous vessels normal in course and caliber.
Bones / soft tissues:	No abnormalities of bones and soft tissues.
Chest:	No abnormalities of the visualized lower portions of lungs and mediastinum.

Impression
- Two hepatocellular carcinomas in segment VIII (3.8 cm) and in segment VI (2.1 cm) (LI-RADS 5).
- Advanced liver cirrhosis with extensive portosystemic collaterals and lienorenal shunt.
- No portal vein thrombosis.
- Extensive cirrhotic ascites in all four quadrants.

____
LI-RADS NC = not categorizable due to image degradation or omission
LI-RADS M = probably / definite malignancy not HCC specific
LI-RADS TIV = definite tumor in vein
LI-RADS 5 = definitely HCC
LI-RADS 4 = probably HCC
LI-RADS 3 = intermediate probability of malignancy
LI-RADS 2 = probably benign
LI-RADS 1 = definitely benign observation

Reference
http://www.acr.org/quality-safety/resources/LIRADS

Figure S1: Side-by-side comparison of free-text and structured report for HCC screening. The structured report on the right side represents a level 1 reporting template according to Nobel et. al (2020). It has a structured layout and predefined subheadings for different anatomic structures, as well as prepopulated normal findings, however the content remains to be editable during report creation.

Eur Radiol (2023) Vosshenrich J, Nesic I, Boll DT, Heye T

Technique
Siemens 3T SkyraFIT MRI. 17 ml Dotarem.

Findings
No comparison exams.

There is a 3.2 cm measuring mildly T2 hyperintense, contrast enhancing polypoid lesion with diffusion restriction in the middle third of the rectum, about 6 cm above the anal verge (Se 4, Im 12). The lesion seems necrotic in the center but there are no signs of an adjacent fluid collection. It involves more than 2/3 of the rectal circumference and extends through the rectal wall into the mesorectal fat but does no reach the mesorectal fascia. Normal appearance of the internal and external sphincter. There are several lymph nodes in the mesorectal fat. Some are mildly enlarged with a short axis diameter of up to 11 mm (Se 6, Im 20) and some of them are round in appearance but not enlarged (e.g. 6 mm, Se 6, Im 23). All of these nodes are restricted in diffusion. The mesorectal vessels appear to be unremarkable.
Bladder and visualized lower thirds of the ureters appear normal. No masses. St.p. hysterectomy. Ovaries normal for age and without evidence of cystic or solid masses. Diverticulosis of the sigmoid colon without active inflammation. The visualized portions of the small bowel are normal. No signs of bowel obstruction. Small amount of fluid in pouch of Douglas, otherwise no evidence of ascites. Moderate degenerative changes of the lumbar spine with moderate canal stenosis at the L4/5 level due to disk protrusion and thickened ligamenta flava. No fracture. No suspicious osseous lesions of the pelvis. Bilateral tendinopathy of the gluteus minimus tendon with corresponding fatty muscle degeneration. Otherwise unremarkable soft tissues.

Impression
- 3.2 cm rectal cancer in the middle third of the rectum with extramural extension.
- Five suspicious lymph nodes in the mesorectal fat.
- No evidence of pelvic metastases.

Technique
Siemens 3T Vida MRI. 18 ml Dotarem.

Findings
No comparison exams.

	**Rectal tumor (T)**
Location:	Middle third of the rectum (6-12 cm above the anal verge)
Distance from anal verge:	7.5 cm
Craniocaudal length:	2.7 cm
Clock face of tumor	3 o'clock to 10 o'clock
Distance to anal sphincter:	4 cm
Relation to peritoneal reflection:	Below
Relation to puborectalis sling:	Above
Depth of infiltration:	T3c/d (>5 mm mesorectal infiltration) at 7 o'clock
Distance to mesorectal fascia:	CRM+ (< 1 mm) at 7 o'clock
Extramural venous invasion:	Yes
	**Regional lymph nodes (N)**
Mesorectal / presacral:	> 3 suspicious mesorectal lymph nodes (N2)
Distance to mesorectal fascia:	CRM- (> 1 mm)
	**Metastasis (M)**
Extra mesorectal lymph nodes:	No suspicious lymph nodes.
Peritoneum:	No evidence of peritoneal carcinomatosis. No ascites.
Pelvic organs:	No evidence of tumor spread or metastases.
Bones:	No evidence of metastasis.
Other findings:	None

Impression:
- Rectal cancer in the middle third with involvement of the mesorectal fascia at 7 o'clock. (T3c/d)
- No involvement of the peritoneal reflection, puborectalis sling or anal sphincter.
- At least six mesorectal lymph node metastases. (N2)
- No evidence of pelvic metastasis (M0)

- <u>Preliminary TNM:</u> T3c/d N2 M0 (CT chest/abdomen/pelvis to complete staging is necessary).

Figure S2: Side-by-side comparison of free-text and structured rectal cancer staging MRI report. The structured report on the right side represents a level 2 reporting template according to Nobel et. al (2020). It incorporates both a structured layout and structured content. During report creation, the radiologist can only choose predefined content options from dropdown menus or enter numeric values.

Supplementary Material

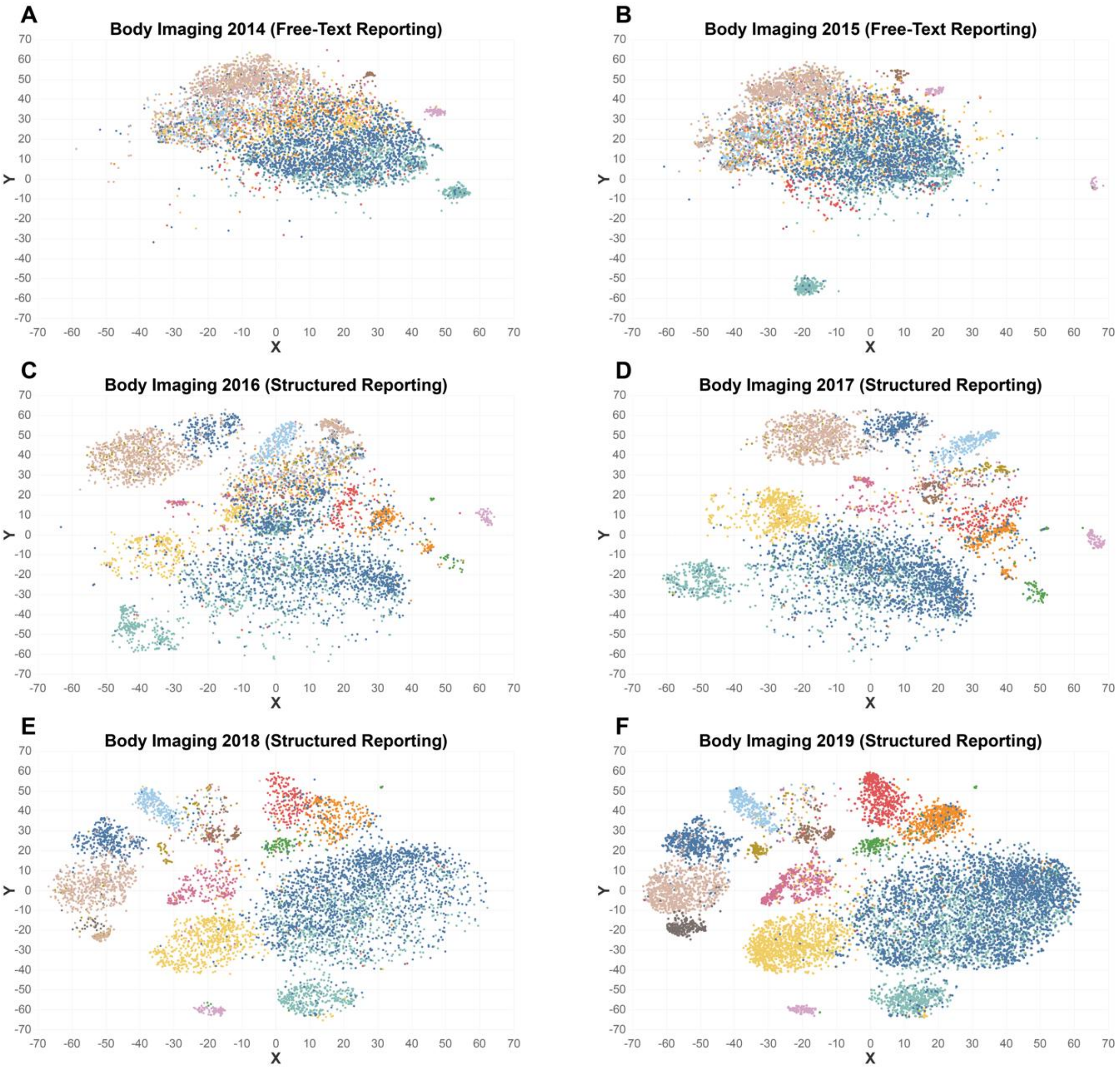

Figure S3: Evolution of radiology report distribution in vector space over a period of six years from (A) 2014 to (F) 2019. With free-text reporting in 2014 (A) and 2015 (B) data points of distinct types of radiology reports overlap and cannot be distinguished based on vector location. Following the introduction of structured reporting templates in 2016 (C) clustering and distinguishability of distinct radiology report types continuously increase (D-F). Color-coding represents distinct report types (e.g. CT abdomen/pelvis).
